# Text messaging reminders for influenza vaccine in primary care: a cluster randomised controlled trial (TXT4FLUJAB)

**DOI:** 10.1136/bmjopen-2015-010069

**Published:** 2016-02-19

**Authors:** Emily Herrett, Elizabeth Williamson, Tjeerd van Staa, Michael Ranopa, Caroline Free, Tim Chadborn, Ben Goldacre, Liam Smeeth

**Affiliations:** 1 London School of Hygiene and Tropical Medicine, London, UK; 2 Health eResearch Centre, Farr Institute, University of Manchester, Manchester, UK; 3 Faculty of Sciences, Division of Pharmacoepidemiology and Clinical Pharmacology, Department of Pharmaceutical Sciences, Utrecht Institute for Pharmaceutical Sciences, Utrecht University, Utrecht, The Netherlands; 4 Health and Wellbeing Directorate, Public health England, Wellington House, London, UK; 5 Department of Primary Care Health Sciences, Centre for Evidence Based Medicine, University of Oxford, Radcliffe Observatory Quarter, Oxford, UK

**Keywords:** PUBLIC HEALTH

## Abstract

**Objectives:**

(1) To develop methods for conducting cluster randomised trials of text messaging interventions utilising routine electronic health records at low cost; (2) to assess the effectiveness of text messaging influenza vaccine reminders in increasing vaccine uptake in patients with chronic conditions.

**Design:**

Cluster randomised trial with general practices as clusters.

**Setting:**

English primary care.

**Participants:**

156 general practices, who used text messaging software, who had not previously used text message influenza vaccination reminders. Eligible patients were aged 18–64 in ‘at-risk’ groups.

**Interventions:**

Practices were randomly allocated to either an intervention or standard care arm in the 2013 influenza season (September to December). Practices in the intervention arm were asked to send a text message influenza vaccination reminder to their at-risk patients under 65. Practices in the standard care arm were asked to continue their influenza campaign as planned.

**Blinding:**

Practices were not blinded. Analysis was performed blinded to practice allocation.

**Main outcome measures:**

Practice-level influenza vaccine uptake among at-risk patients aged 18–64 years.

**Results:**

77 practices were randomised to the intervention group (76 analysed, n at-risk patients=51 121), 79 to the standard care group (79 analysed, n at-risk patients=51 136). The text message increased absolute vaccine uptake by 2.62% (95% CI −0.09% to 5.33%), p=0.058, though this could have been due to chance. Within intervention clusters, a median 21.0% (IQR 10.2% to 47.0%) of eligible patients were sent a text message. The number needed to treat was 7.0 (95% CI −0.29 to 14.3).

**Conclusions:**

Patient follow-up using routine electronic health records is a low cost method of conducting cluster randomised trials. Text messaging reminders are likely to result in modest improvements in influenza vaccine uptake, but levels of patients being texted need to markedly increase if text messaging reminders are to have much effect.

**Trial registration number:**

ISRCTN48840025.

**Strengths and limitations of this study**

- Text messaging is already commonly used in general practice to remind patients about receiving their influenza vaccine, but this study is the first to estimate the effectiveness of this intervention.
- This cluster randomised trial in English primary care recruited and randomised 156 general practices, including over 100 000 at-risk patients.
- The proportion of patients whose mobile telephone numbers are recorded by their general practice is low, limiting the power of our study to detect an effect.
- Using routinely recorded electronic health records to ascertain outcome data was a novel, low-cost method of trial data collection.

## Introduction

Annual seasonal influenza vaccination is offered to certain patient groups in the UK, including patients aged 65 years and older, and those who are aged under 65 years with one or more chronic conditions (the ‘at-risk’ under 65s). The UK government sets targets for vaccine uptake in these groups; in the 2013/2014 influenza season the target was 75%.^1^ While uptake in the over 65s reaches this target in many practices, barriers to vaccination result in unmet targets for the at-risk under 65s (uptake in 2012/2013 was 49.6%).^2^ These barriers include failure of healthcare workers to recommend vaccination, low awareness of eligibility for vaccination, perceptions about severity of influenza, and personal beliefs about vaccine effectiveness, pain and side effects.^3–5^ In the face of these barriers, strategies to improve vaccine uptake are required.

Vaccination reminders by letter, postcard and telephone have shown to be effective,^6^ ^7^ but these interventions become costly when there are large numbers of at-risk patients. Text messaging is a useful tool because it is cheap, fast and personal, and because mobile phone ownership in the UK is widespread (94% of adults own a mobile phone).^8^ Consequently, text messaging has been increasingly used in the National Health Service to contact patients for appointment reminders and other health-related issues. In the 2010–2011 influenza season, text messages were used in roughly a third of practices for influenza vaccination reminders^9^ but there is no evidence that this strategy increases uptake in primary care.

An emerging trial methodology utilises routinely collected electronic health record sources to ascertain outcome data, at far lower cost than traditional methods of data collection.^10^ The feasibility of this approach in primary care practices requires further examination. Therefore we performed a cluster randomised trial in English primary care, embedded within routine electronic health records, to determine the effectiveness of text message influenza vaccination reminders in increasing influenza vaccination uptake among the at-risk under 65s. Randomising individuals within practices would have been logistically complex and so a cluster design was chosen to reduce the burden to practices of participating in our trial. The aims of the study were (1) to develop the methods for conducting cluster randomised trials of text messaging interventions, and to evaluate feasibility of recruitment, identification of eligible practices and patients, and to follow-up through routine electronic health records at very low cost; and (2) to implement a cluster randomised trial to test the effectiveness of using a text messaging influenza vaccine reminder in achieving an increase in practice-level influenza vaccine uptake in patients aged 18–64 years with chronic conditions, compared to standard care.

## Methods

Further details of our methods have been described previously in our published protocol,^11^ and the formal study protocol is available in online supplementary appendix 1.

### Trial design

This was a two arm, pragmatic, cluster randomised trial, with general practices as clusters allocated in a 1:1 ratio to each arm.

### General practice and patient eligibility

The study took place in the 2013/2014 influenza season (vaccinations September–December) among English primary care practices in three settings: (1) the Clinical Practice Research Datalink (CPRD),^12^ a primary care database based on Vision software and covering 8% of the UK population; (2) TPP SystmOne software^13^ users; (3) iPLATO text messaging software users in London^14^ (initially only practices in Islington and Barnet were targeted, and this was later extended to all London boroughs to achieve the required sample size).

Practices were recruited between July 2013 and October 2013. Practices were eligible if they already used text messaging software and did not use text messaging to contact patients about influenza vaccine in the 2012/2013 season. Eligibility of CPRD practices was determined based on the routinely recorded data and only practices meeting the criteria were invited. All users of iPLATO across London boroughs, and all practices using TPP SystmOne software were invited to the trial and eligibility was determined at the next stage.

Within participating practices, eligible patients were those aged 18–64 years in at-risk groups due to chronic conditions at the start of the influenza vaccination season. This comprises patients who are under 65 with chronic heart disease, chronic neurological disease, diabetes, chronic kidney disease, chronic liver disease, chronic respiratory disease and immunosuppression, as set out by the Chief Medical Officer.^15^ Pregnant women and carers, who are also eligible to get the vaccine, were not targeted in our study to avoid ethics concerns (to avoid contacting patients by text message whose circumstances are time dependent and may have changed without being updated in the medical record). Practices are able to identify these individuals as part of routine care, as these patients are targeted for seasonal influenza vaccination. Patients were excluded from the study if they transferred out of the practice or died before the end of data collection (31 December 2013). Consent was gained from the general practitioner at each general practice, before randomisation had taken place. Patients did not provide consent to be part of this trial. The trial co-ordinator enrolled general practices and informed the practices of their allocation.

### Interventions

Practices were allocated to either a standard care arm or an intervention arm. In the standard care arm, practices were asked to continue with their seasonal influenza vaccination campaign as planned, typically using measures such as posters in the practice and letters to patients. In addition to their planned campaign, practices in the intervention arm were asked to send a text message influenza vaccination reminder using their in-practice text messaging software, to patients aged 18–64 years in at-risk groups. This required practices to perform two steps: (1) identify eligible patients: most clinical software systems have algorithms to identify eligible patients and practices were encouraged to use these; (2) send a tailored text message to these patients using software embedded in the electronic health record.

The recommended text message content was “Hello PATIENT NAME, to reduce your risk of serious health problems from flu, we recommend vaccination. Call PRACTICE PHONE NUMBER to book. PRACTICE NAME.” The message content was designed by behavioural specialists and detail is given in the study protocol.^11^ Step by step instructions were provided (see online supplementary appendix 2) but no formal training was given to practices as they were included in the trial based on their experience in using text messaging for other purposes (eg, appointment reminders).

Practices were instructed to send one text message to their patients at the start of their seasonal influenza campaign, but were free to send additional messages later in the season if they chose to. Details of the intervention delivery, including which member of staff sent the text message, the time of day the messages were sent, and whether messages were sent in bulk or individually were left to the discretion of the practice (though guidance was provided in the step by step instructions).

### Randomisation, allocation concealment and blinding

After enrolment to the trial, practices were randomly allocated (1:1) to the standard care arm or the intervention (influenza text messaging reminder) arm. Randomisation was performed within strata defined by the clinical software system or text messaging software used by each general practice (TPP SystmOne, CPRD, iPLATO), and was additionally stratified by region (for CPRD practices) and borough (for London-based practices). Block randomisation, using block sizes of 2, 4 and 6, was performed. The allocation sequence was generated by an independent statistician who was blind to practice name. The trial co-ordinator, who enrolled the practices and informed them of their allocation, was unaware of the allocation until a complete block had been randomised. It was not possible to blind practices to their allocation but data management and analysis were performed blind to allocation.

### Outcomes

The primary outcome, measured at the cluster level (GP practice), was influenza vaccination uptake among patients aged 18–64 years in the seven prespecified risk groups during the period between 1 September and 31 December 2013. This time period would have captured the majority of influenza vaccinations.

Secondary outcomes were (1) the proportion of eligible patients sent a text message, and (2) outcomes used to establish the feasibility of our methods, including the rate of recruitment, adherence to the text messaging protocol, adverse events and problems with message delivery, availability of electronic health record data, and the time and cost required to gather data.

### Data collection

Data on vaccination uptake, overall and by clinical risk group, were obtained through extraction of routinely collected data. Additional consent from GPs was required for further demographic patient-level data (age and sex) and so were only available for a subset. In practices that contribute data to the CPRD or use TPP SystmOne software, relevant patient-level data were extracted from the database using prespecified Read codes (the standard clinical terminology system used in general practice in the UK). Within London practices using iPLATO software, data were extracted by the clinical software supplier of the practice or the practices themselves. Data extracted included patient registration details, age, sex, death date, clinical risk group, vaccination status and influenza vaccine invites. Codes indicating risk groups and vaccinations were identified using PRIMIS Plus Read code lists, available online.^16^ In one of the three settings, where text messaging was systematically recorded in the practice electronic health record, adherence to the intervention was assessed by study investigators. It was not possible to measure adherence to the exact wording of the text message. All data were stripped of personal identifiers before being supplied to the study team. Where patient-level data were not available, practice-level data were supplied by Immform, the UK's surveillance system for influenza vaccine uptake.^17^

### Sample size

To achieve 90% power with a 5% significance level, assuming an intracluster correlation of 0.024, 100 practices were required to identify a 7.5% relative increase in vaccine uptake from 54% to 58%.^18^ To account for differences in the number of eligible patients per practice and dilution of the intervention effect through potential contamination between arms, we chose to recruit and randomise 150 practices to the study.^11^

### Statistical methods

The primary analysis was by intent to treat, comparing the cluster-specific proportions of vaccine uptake between randomised arms using a two-sided t test, with cluster proportions weighted to account for differing numbers of at-risk patients across practices. Minimum-variance weights were used,^19^ which weight cluster i by 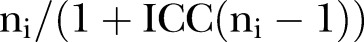 where ICC is the intracluster correlation coefficient and n_i_ is the number of at-risk patients in that cluster.

#### Sensitivity and subgroup analyses

We conducted a number of secondary analyses to account for contamination between the randomised arms. First, we used an instrumental variables approach to fit a structural mean model, using the minimum-variance weights from the primary analysis, to estimate the intervention effect under hypothetical full adherence,^20^ that is, assuming all eligible patients in the active arm received an influenza text message, and no eligible patients in the other arm received a text message. We additionally performed a per-protocol analysis which restricted analysis to the patients in each practice who actually received their allocated intervention. These analyses were restricted to practices for which data were available regarding text messaging.

In a non-randomised preplanned comparison, we explored whether the intervention effect differed by time of day the text message was sent by using a multilevel logistic regression model including a random main effect for practice fitted to patient-level data. These analyses were also restricted to practices for which data were available regarding text messaging.

A post hoc exploratory analysis was conducted using similar multilevel logistic regression models to assess the intervention effect within seven predefined at-risk patient subgroups.

To assess whether participation in the trial changed vaccine uptake (Hawthorne effect), we compared practices taking part in the trial to those outside the trial using the ResearchOne primary care database (a research database holding over 400 TPP SystmOne practices). T tests were used to compare the at-risk population size and vaccine uptake of standard care practices taking part in the trial to practices outside the trial. Data were analysed using Stata V.14.1.

#### Missing data

Receipt of in-practice influenza vaccine was automatically recorded in the clinical system from which we extracted the data, so there was no missing outcome data. However, if patients received their influenza vaccine outside the practice, this would not have been recorded unless the patient informed their GP. Patients transferring out of the practice or dying during the study period were not included in the denominator. Adherence to the intervention could not be measured for all practices because specific vaccine reminder text messages were systematically recorded for practices using only TPP SystmOne clinical software.

### Substudy

A substudy was conducted among two intervention practices, whose administrative staff sent a short questionnaire (four questions) to patients who had received the text message. Questionnaires were sent after the data collection phase for the trial was complete (December 2013). Patients were asked to return anonymised responses directly to the study team by mail (postage pre-paid) via a self-completion questionnaire (free of any patient or practice identifiers) about their memory of having received the text message, any objections to the message, and whether it encouraged them to receive the vaccine (Questionnaire in online supplementary appendix 3).

### Practice questionnaire

A questionnaire was sent to all practices in the intervention group to identify comments and concerns from practice staff regarding use of the text message (see online supplementary appendix 4). The questionnaire was sent electronically to the staff member who had been the named contact for the trial, with practice staff members able to complete online, and was sent in early December, when practices were nearing the end of the flu vaccination season.

This trial was approved by Surrey Borders Ethics Committee (13/LO/0872).

## Results

**Figure 1:**
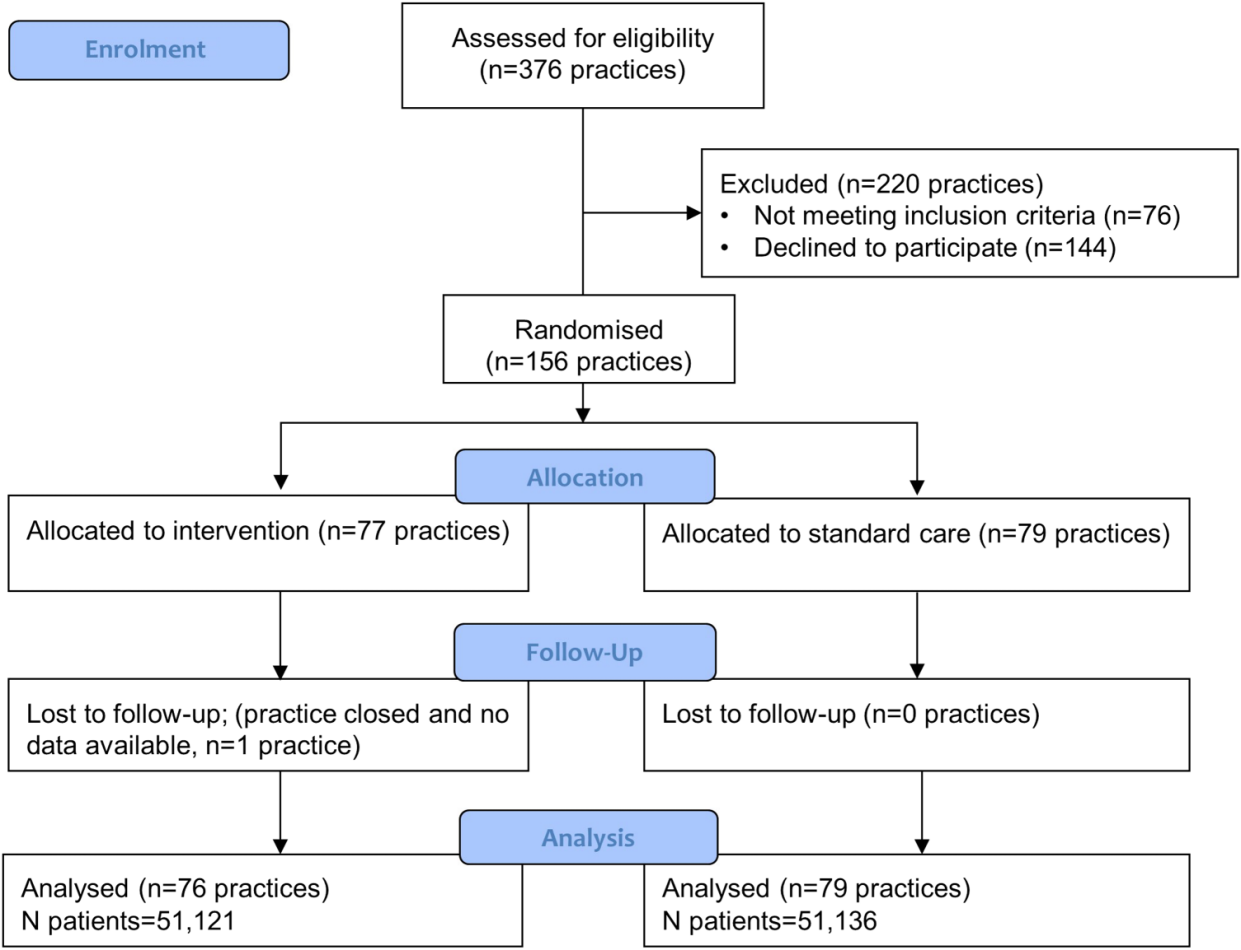
Consort flow diagram.

Our invitation to the trial was sent to over 2600 English practices through the Primary Care Research Network, TPP SystmOne practices, the CPRD and users of iPLATO text messaging software. 376 expressed an interest in the trial and 156 (41%) were eligible and provided consent (figure 1). One hundred and fifty-six practices participated in the study. Seventy-seven were allocated to the intervention group and 79 to standard care (figure 1). Practices were followed up for text messaging and influenza vaccine uptake between 1 September 2013 and 31 December 2013. Data for the primary outcome were available in 155 of 156 practices. One intervention group practice was lost to follow-up due to practice closure. Additional demographic patient-level data, including age and sex, were available in 144/156 practices (92%, 73/79 standard care, 71/77 intervention). Overall, the final trial analysis included 102 257 at-risk patients aged 18–64 years (table 1).

**Table 1:** Distribution of clusters and eligible patients in the standard care and intervention arms of the trial

	Standard care	Intervention
N clusters	79	76
Region (n clusters)		
South East	4	3
London	18	16
East of England	17	16
South West	9	8
West Midlands	1	3
East Midlands	12	12
North East and North West	4	3
Yorkshire and the Humber	14	15
Clinical software or iPLATO user		
CPRD	1	2
TPP SystmOne	61	59
iPLATO	10	10
N patients at-risk	51 136	51 121
Median (minimum, maximum) at-risk patients per cluster	583 (125, 1678)	637 (79, 3022)
Sex, n (%)*		
Men	24 420 (51.2)	24 182 (51.2)
Women	23 285 (48.8)	23 005 (48.8)
Age group, n (%)*		
18–34	8113 (17.0)	8216 (17.4)
35–50	16 197 (34.0)	15 797 (33.5)
51–64	23 395 (49.0)	23 174 (49.1)
Risk group, n (%)†		
Chronic heart disease	8419 (16.5)	8291 (16.2)
Diabetes	12 999 (25.4)	13 370 (26.2)
Chronic respiratory disease	24 244 (47.4)	24 393 (47.7)
Chronic liver disease	1728 (3.4)	1605 (3.1)
Chronic kidney disease	3190 (6.2)	3045 (6.0)
Chronic neurological disease	5949 (11.6)	5853 (11.4)
Immunosuppression	3341 (6.5)	3766 (7.4)

*From patient-level data, based on 145 practices (N=94 892; 47 705 standard care, 47 187 intervention).

†Groups not exclusive.

CPRD, Clinical Practice Research Datalink.

### Primary outcome

In the standard care arm of the trial, mean vaccine uptake across practices was 50.7% and in the intervention arm was 52.4%. A minimum variance weighted t test showed that there was an absolute 2.62% (95% CI −0.09% to 5.33%) increase in practice-level vaccine uptake among at-risk patients aged 18–64 years (two-sided p value=0.058) in the text messaging intervention group, compared to standard care (table 2, observed ICC=0.029). This corresponds to a relative increase of 5.17%. In a sensitivity analysis, further adjustment for clinical software system showed a similar absolute increase in vaccine uptake of 2.66% (95% CI −0.03% to 5.34%, p=0.052). Based on this analysis, the number needed to text (NNT) to achieve one additional influenza vaccination is 38.2 (95% CI −1.01 to 77.4).

**Table 2:** Number of practices, patients and estimates of effectiveness for the text message influenza vaccine reminder

	Standard care	Intervention
Practice-level summary		
Number of practices	79	76
Per cent vaccinated in cluster—median (minimum, maximum)	50.6 (27.7 to 76.2)	53.4 (38.5 to 81.1)
Per cent vaccinated in cluster—mean (SD)	51.7% (8.8%)	54.3% (8.4%)
Patient-level summary		
Total patients	51 136	51 121
Number vaccinated	25 939 (50.7%)	26 804 (52.4%)
Estimated difference in uptake percentage:		
Primary analysis*	2.62 (95% CI −0.09 to 5.33),	p=0.058
Sensitivity analysis, further adjusting for data source†	2.66 (95% CI: −0.03 to 5.34)	p=0.052

*Minimum variance weighted cluster t test.

† Data sources: CPRD, TPP SystmOne, EMIS/Vision; used to stratify randomisation.

CPRD, Clinical Practice Research Datalink.

#### Efficacy and per protocol analyses

Only TPP SystmOne practices recorded text messaging systematically in a way that could be extracted from the electronic health record. Among these practices (58 intervention, 58 standard care), in the intervention arm, 6 of 58 practices (10%) did not send any text message to at-risk patients. In the standard care arm, 21 of 58 practices (36%) sent a vaccine reminder text message to at least one patient. Among intervention practices, the majority of messages were sent in September/October (96%), with 4% sent in November/December. Among the intervention practices, the median proportion of patients sent a text message was 21.0% (IQR 10.2% to 47.0%), representing 156 messages per practice (IQR 65 to 287) (it was not possible to measure the proportion of patients whose phone number was held by the practice) (table 3). In the standard care arm, the median proportion was 0% (IQR 0–6.3%), corresponding to a median of zero messages per practice (IQR 0–61). Characteristics of patients who were sent a message are described in table 4.

**Table 3:** Summary of adherence to randomised allocation in 116 TPP SystmOne subset of practices

	Standard care	Intervention
Practice-level summary		
N practices	58	58
At least 1 influenza vaccine reminder text message sent—N practices (%)	21 (36.2)	52 (89.7)
Percentage of eligible patients texted—N practices (%)		
None	37 (63.8)	6 (10.3)
1% to <10%	7 (12.1)	8 (13.8)
10% to <20%	4 (6.9)	14 (24.1)
20% to <50%	7 (12.1)	18 (31.0)
50% to <75%	2 (3.5)	10 (17.2)
75% to 100%	1 (1.7)	2 (3.5)
Percentage of eligible patients sent a message—median (IQR)	0 (0–6.3)	21.0 (10.2–47.0)
Number of messages sent—median (IQR)	0 (0–61)	155.5 (65–287)
Number of messages sent—mean (SD)	45.3 (84.9)	191.6 (164.2)
Patient-level summary		
Number (%) of patients sent a message	2628 (6.5%)	11 113 (27.1%)

**Table 4:** Text messages sent to patients in the standard care and intervention arms of the trial, by clinical risk group and demographic characteristics

	Standard care		Intervention	
	N in group	Sent text message n (%)	N in group	Sent text message n (%)
**N**	43 143	2732 (6.3)	44 037	11 515 (26.1)
Clinical risk group*				
Chronic heart disease	6651	436 (6.6)	6657	1698 (25.5)
Diabetes	2367	136 (5.7)	2387	630 (26.4)
Chronic respiratory disease	1251	66 (5.3)	1182	321 (27.2)
Chronic liver disease	4571	252 (5.5)	4583	1030 (22.5)
Chronic kidney disease	20 240	1187 (5.9)	20 703	5462 (26.4)
Chronic neurological disease	10 120	999 (9.9)	10 318	3807 (36.9)
Immunosuppression	2343	87 (3.7)	2341	490 (20.9)
Age group*				
18–34	6823	426 (6.2)	7050	1948 (27.6)
35–50	13 809	958 (6.9)	13 598	4147 (30.5)
51–64	20 001	1244 (6.2)	22 425	5018 (24.6)
Sex*				
Male	20 752	1464 (7.1)	21 012	5777 (27.5)
Female	19 881	1164 (5.9)	20 061	5336 (26.6)

*Available in patient-level data from TPP SystmOne users only (N=116 practices, 81 706 patients; 40 633 standard care, 41 073 intervention).

In secondary analyses among practices where text messages were systematically recorded (TPP SystmOne n=116 practices), instrumental-variables-based efficacy analyses demonstrated that texting 100% of eligible patients, compared with texting 0%, could achieve a 14.3% (95% CI −0.59% to 29.2%) increase in vaccine uptake. Based on this analysis, the NNT=7.0 (95% CI −0.29 to 14.3). A more modest aim of texting 50% of patients could achieve a 7.2% (95% CI −0.30% to 14.6%) increase in vaccine uptake.

A per protocol analysis using the same minimum variance weighted t test, but excluding patients who did not receive their allotted intervention, showed an absolute increase in vaccine uptake of 9.40% (95% CI 4.68% to 14.1%) in the text messaging intervention group, compared to standard care.

#### Subgroup analyses: among risk groups

Post hoc exploratory analysis using mixed models demonstrated that, overall, the odds of vaccination increased by 12% (OR=1.12, 95% CI 1.00 to 1.25) in the text messaging arm of the trial compared to standard care. Within the seven risk groups (chronic heart disease, diabetes, chronic respiratory disease, liver disease, chronic kidney disease, chronic neurological disease, immunosuppression) there was little variation in the ORs; text messaging appears to work better in patients with chronic heart disease (OR=1.19, 95% CI 1.05 to 1.34, p for interaction comparing the OR for text messaging between patients with and without chronic heart disease=0.036), though less well in patients with diabetes (OR=1.04, 95% CI 0.93 to 1.17, p for interaction=0.011) (table 5).

**Table 5:** Vaccination uptake and intervention effectiveness by subgroups of clinical risk group, age group and sex

	Standard care		Intervention		
	N in group	Vaccinated n (%)	N in group	Vaccinated n (%)	OR (95% CI)
**N**	51 136	25 939 (50.7)	51 121	26 804 (52.4)	1.11 (1.00 to 1.25)
Clinical risk group					
Chronic heart disease	8419	4409 (52.4)	8291	4592 (55.4)	1.19 (1.05 to 1.34)
Diabetes	12 999	8968 (69.0)	13 370	9294 (69.5)	1.04 (0.93 to 1.17)
Chronic respiratory disease	24 244	11 767 (48.5)	24 393	12 180 (49.9)	1.12 (1.00 to 1.25)
Chronic liver disease	1728	730 (42.2)	1605	700 (43.6)	1.11 (0.94 to 1.33)
Chronic kidney disease	3190	1727 (54.1)	3045	1722 (56.6)	1.14 (0.98 to 1.32)
Chronic neurological disease	5949	2906 (48.8)	5853	2979 (50.9)	1.14 (1.00 to 1.30)
Immunosuppression	3341	1686 (50.5)	3766	2029 (53.9)	1.19 (1.03 to 1.37)
Age group*					
18–34	8113	2574 (31.7)	8216	2755 (33.5)	1.16 (1.01 to 1.32)
35–50	16 197	7344 (45.3)	15 797	7382 (46.7)	1.11 (0.98 to 1.25)
51–64	23 395	14 255 (60.9)	23 174	14 423 (62.2)	1.10 (0.98 to 1.24)
Sex*					
Male	24 420	11 876 (48.6)	24 182	12 208 (50.5)	1.12 (1.00 to 1.26)
Female	23 285	12 297 (52.8)	23 005	12 352 (53.7)	1.09 (0.97 to 1.23)

*Available in patient-level data only (N=145 practices, 101 159 patients; 50 659 standard care, 50 500 intervention).

#### Subgroup analyses: by age and sex

Post hoc exploratory analyses showed no differences in the effect of the intervention by age group or sex. Results are shown in table 5.

#### Timing of text message

In a post hoc non-randomised analysis, compared to patients for whom no message was sent, those receiving a text message at any time of day had increased odds of being vaccinated. The estimated effect of sending messages in the morning appeared to be slightly lower than for other times of day, and the effect in the evening appeared to be slightly higher (morning, up to midday OR=1.18 (1.10 to 1.26); early afternoon 12:00–15:30 OR=1.40 (1.32 to 1.50); late afternoon 15:30—18:00 OR=1.29 (1.18 to 1.42); evening 18:00 onwards OR=1.44 (1.09 to 1.89), p for interaction between times of day=0.002) (see online supplementary table S1).

#### Hawthorne effect

Among practices in ResearchOne, we compared practices in the standard care arm of the trial (n=55) to those outside the trial (n=281) in terms of influenza vaccine uptake. This showed no difference in uptake (uptake in trial 50.0%, uptake outside trial 50.8%, p=0.53), though practices taking part in the trial tended to have slightly larger at-risk populations than those not taking part (mean practice at-risk population in trial 705 compared to 574 outside the trial, p=0.02).

### Secondary outcomes

Interest in the trial was higher than anticipated, but our inclusion criteria prevented 76 (20%) of interested practices from taking part. Among all practices invited, the proportion recruited was roughly 6%.

Practices in the trial understood the randomisation process, but there was some contamination between trial arms (table 3).

The patient substudy and practice questionnaire allowed us to monitor adverse events and harms to patients. Of 77 intervention group practices, 72 (94%) responded to the practice questionnaire. Three practices (4.2%) reported difficulties in sending the text message; one because of a failure of the text messaging software, one because ineligible patients were accidentally selected to receive the message, and one because only small numbers of patients had consented to receive messages.

Five practices (6.9%) reported complaints from patients about the message (though numbers of complaints were not recorded): due to ineligible patients receiving messages (n practices=1), receipt of >1 message (n=1); receipt of a reminder after making an appointment (n=1), and two resulting from protocol deviation (text message was missing the recipient's name or message contained incorrect clinic times). Sixty-two practices (86.1%) reported that text messaging for influenza vaccine reminders was worthwhile. No unintended effects were reported to the study team.

Eight hundred and twenty-five patients were invited to complete the substudy questionnaire. One hundred patients responded (response rate 12%). Three quarters (75/100) recalled receiving the text message. Of those that recalled the message, 4 (5.3%) objected to the message with no reason given, but 48 (64.0%) reported being encouraged by the text message to make an appointment for their vaccine.

A final outcome was to evaluate the feasibility of ascertaining practice data regarding text message delivery and influenza vaccine uptake. Changes were made to the methods for ascertaining outcome data in response to difficulties in acquiring outcome data via the planned routes. One practice withdrew from the trial due to closure during data collection. For the remaining 155 practices, access to data from existing primary care databases (practices in CPRD and ResearchOne, the primary care database of over 400 TPP SystmOne practices) was straightforward. These databases provided data for 111 practices. However, ascertaining the data was more expensive and logistically complex than expected for iPLATO software users and among practices that did not consent to join the CPRD and ResearchOne databases (N=44 practices; 21 intervention group). For these practices, access to data was possible directly through TPP SystmOne, and with some additional costs data were accessed through clinical software providers EMIS and Vision (see online supplementary table S2). Through these routes (with full practice consent), full patient-level data were available from 144 practices.

Data from 11 practices were available only at practice-level, and after gaining practice consent, this was provided through Immform.^17^ This allowed assessment of the primary outcome (vaccine uptake) among subgroups of risk but contained no information on text messaging, age or sex. Additionally, in most data sources, text messaging is not recorded systematically, meaning that we were only able to perform efficacy and per-protocol analyses among practices using TPP SystmOne software. The additional costs of data collection raised the cost of the trial substantially but the total cost of the trial was less than £1 per patient. A breakdown of costs is given in table 6.

**Table 6:** Breakdown of trial costs for TXT4FLUJAB

	Cost in GBP (£)
Incentive payments for intervention group	15 600.00
Invitation letters, postage, administration	3700.00
iPLATO software support	1200.00
Planned data costs	3500.00
Research coordination and analysis	50 000.00
Unplanned data costs	20 600.00
Substudy cost	2770.00
Trial sponsorship	1000.00
Total	98 370.00

## Discussion

### Statement of principal findings

Embedding a trial within existing, routinely recorded primary care electronic health records was a feasible, efficient and low-cost approach. Practices allocated to the text messaging intervention arm had higher influenza vaccine uptake among at-risk patients aged 18–64 years than practices allocated to standard care. However, there was only weak evidence of an effect and the increase may have been due to chance. In most practices, adherence to the intervention was low and messages were sent to an average of one-fifth of eligible patients. An efficacy analysis indicated that if practices sent a reminder text message to 50% of at-risk patients, vaccine uptake could increase by an absolute proportion of 7%, while texting 100% of patients could achieve a 15% increase in uptake. This indicates that the modest intervention effect observed may be partly attributable to low adherence. Participating practices tended to have larger at-risk populations than practices outside the trial. The standard care arm had the same vaccine uptake as practices not in the trial, indicating that our results are likely to be generalisable to all practices in England. Recent evidence has shown that text messaging is effective in other contexts,^21^ ^22^ and our trial recruitment phase, questionnaire and substudy indicated the popularity of text messaging among practices and patients. Our trial provided some evidence that this intervention is likely to result in modest improvements in influenza vaccine uptake among at-risk groups aged 18–64 years.

### Strengths

This study demonstrates the feasibility of embedding a trial within routinely recorded primary care electronic health records. Recruitment was feasible with the support of the NIHR Primary Care Research Networks. Our method was advantageous for two reasons: first, it minimised the burden of research for practices and encouraged recruitment, and second, it reduced the cost compared to trial-specific data collection. We have established the feasibility of recruiting practices (beyond initial expectations), randomising practices to a text messaging intervention and ascertaining outcome data using the electronic health record. Our intervention was simple and easy for practices to implement and this was reflected in the positive feedback that we received from practices in the text messaging group. Very few patients expressed annoyance at receiving the message, and while there may have been a response bias in our substudy results, we believe that those who did not respond are less likely to have concerns. One of the key measures of feasibility was our ability to obtain outcome data from practices and, unfortunately, concern over data sharing, particularly in response to care.data,^23^ brought challenges to outcome ascertainment. We obtained patient-level data for 144 of 156 practices and primary outcome data for 155. In doing so we have identified that the optimal method for trials attempting to use electronic health record data is to use only practices with established data extraction procedures. Our trial was a cost-effective approach to reach a population of over 100 000 at-risk patients, costing less than £1 per patient. Further cost-savings would have been possible by using only practices in established research databases.

### Limitations

#### Adherence to the intervention

There was some contamination between the intervention and standard care arms: roughly one-third of standard care practices chose to send a message and one-tenth of intervention practices failed to send a message. This reduced the power of our trial to identify a difference between groups; financial incentives to practices for adherence to the intervention may have been beneficial to reduce contamination. In intervention group practices, an average of one-fifth of patients were sent a message. This was low because not all patients have a mobile phone and many do not give their numbers to their general practice, meaning that the pool of eligible patients that could receive the intervention was smaller than expected. One of the strengths of the trial was that it was restricted to practices that already used text messaging for other purposes, so the proportion of the patient population with a mobile phone number recorded would have been higher than texting-naïve practices. However, it was not possible to measure the proportion of patients for whom a valid mobile phone number was held. A common issue for practices wishing to use text messaging is consent. At the time of the trial, there were no laws in place about gaining consent from patients about text messaging. The NHS has produced guidance for text messaging,^24^ though it does not differentiate between the two models of consent available to practices: (1) an ‘implied consent’ or ‘opt-out’ approach, in which the practice assumes consent for those patients who have provided a mobile telephone number; practices using the ‘implied consent’ approach must have procedures in place to ensure that they meet the NHS Confidentiality Code of Practice^25^; (2) an ‘explicit consent’ or ‘opt-in’ approach, in which each patient must provide explicit consent to receive text messages, even after they have supplied their number. The decision about consent is therefore not straightforward but the Medical Protection Society now recommends that practices should not assume consent and should ask for it explicitly.^26^ Therefore the proportion of patients who can be sent text messages may not increase at a fast pace.

No data were available on the proportion of patients that received or read the text message. Our efficacy analysis assuming intervention adherence of 100% showed that sending text message vaccine reminders could achieve an increase in vaccine uptake as high as 14%. However, the response to a text message reminder among patients who have not provided a mobile telephone number to their general practice may not be the same as those who have already given their number.

As practice staff members identified at-risk patients and sent the text message using in-practice software, we were unable to control adherence to the intervention. Though we were unable to assess the wording of the text message (as this is not routinely recorded by practices) several practices reported modification of the message to suit vaccination clinic times. We believe that the disadvantages of our approach to implementation are outweighed by the advantages of the pragmatic design and cost-savings.

### Comparison to other literature

This is the first study to evaluate the use of text messaging for vaccination reminders in English primary care. In the USA, an individually randomised controlled trial in a low-income population evaluated the use of text messaging reminders sent to parents about their child's influenza vaccinations. This study showed that text messaging was responsible for a 9% relative increase in vaccine uptake.^27^ Elsewhere, text messaging has proven effective in reducing non-attendance for appointments compared to no intervention,^28^ to aid smoking cessation and to improve adherence to antiretroviral treatment.^29^ There is also good evidence that influenza vaccination reminders (letters, postcards and phonecalls) are effective at improving uptake.^7^ However, when compared against other reminder types, a systematic review showed no benefit of text messaging for appointment attendance.^28^

Based on this evidence, we believe that text messages are likely to have some effect on vaccination uptake, but our ability to detect an effect was limited by non-adherence and the following two factors: (1) the practices in our trial were instructed to carry out their seasonal influenza vaccine strategy as planned, with the intervention group additionally asked to send a text message to their at-risk patients. If practices were already devoting resources to their influenza campaigns (eg, emails, letters, phone calls, face-to-face reminders), an additional text message may not be expected to substantially improve uptake. We were unable to assess the effect of other reminder methods as they were not routinely or systematically recorded in the patient electronic health record. (2) While our message addressed some key barriers to vaccination among the at-risk groups (recommendation from general practitioner, severity of influenza, patient's susceptibility to influenza, prompt for patients that may simply have forgotten),^3–5^ there may be remaining barriers that a single text message is unable to overcome, for example, our message would have no effect on patients that are unable to attend clinic times, patients who are concerned about vaccine side effects, or patients who simply do not wish to be vaccinated. These remaining barriers limit the potential of text messaging to improve uptake. These factors may have contributed to the modest intervention effect observed in our study.

There has recently been a move towards more efficient randomised trial designs gathering follow-up data at lower cost from existing data sets. For example: the TASTE trial examined the effect of thrombus aspiration before percutaneous coronary intervention (PCI), using the existing Swedish Coronary Angiography and Angioplasty Registry for follow-up data;^30^ the SAFE-PCI trial compared the safety of radial or femoral access for PCI using the existing National Cardiovascular Data Registry CathPCI Registry (although this trial was abandoned for futility due to low event rates);^31^ ^32^ while two low cost pragmatic randomised trials in the UK used routinely collected NHS primary care electronic health records for outcome data, RETRO-PRO comparing atorvastatin against simvastatin,^10^ and eLung comparing immediate or deferred antibiotics for mild to moderate exacerbation of COPD.^10^ Our study, albeit a cluster randomised trial, was by far the cheapest of any such low-cost pragmatic trial published to date. An overview of the costs in this trial is presented in table 6. Our trial was restricted to practices that were existing users of text messaging and for practices that have not used texting before, there may be some set-up costs for text messaging software. However, these costs do not apply for all software systems and are often borne by the local Clinical Commissioning Group, meaning that the intervention remains cheap at the practice level.

### Implications

Text messaging is a low-cost intervention, popular with patients and quick for practice staff to implement. As practices are incentivised to vaccinate clinical risk groups as part of the Quality Outcomes Framework (QOF) (those with COPD, coronary heart disease, cerebrovascular disease and diabetes), and as part of the NHS Enhanced Service,^33^ a low-cost intervention that may help to increase uptake is likely to have wide appeal. While our trial provides no definitive conclusion about the effectiveness of text message influenza vaccine reminders, it does show that most practices are only able to send text messages to a minority of patients within their practice. If text messaging is to replace invitation letters, or be used as an additional strategy to improve vaccine uptake, then levels of patients being texted would need to markedly increase in order to have an important effect. Use of text message technology is increasing and we have provided some evidence for its utility for increasing vaccine uptake among at-risk groups under 65. We have also demonstrated that cluster randomised trials using routinely collected electronic health record data can be conducted at very low cost.

## Conclusion

This trial demonstrated the feasibility of text messaging interventions for cluster randomisation within English general practice. Owing to the growth of technology and mobile phone usage, we recommend further research to evaluate whether repeated or personalised messages are effective at increasing vaccination uptake in those aged 18–64 years at-risk, and strategies to increase the number of eligible patients with mobile phone numbers recorded. Given the low cost of this trial (<£1 per patient), the novel trial methods used here could be applied to a wide variety of questions relevant to the National Health Service.
